# Prevalence of eosinophilic, atopic, and overlap phenotypes among patients with severe asthma in Saudi Arabia: a cross-sectional study

**DOI:** 10.1186/s12890-022-01856-9

**Published:** 2022-02-17

**Authors:** Hamdan Al-Jahdali, Siraj Wali, Amr S. Albanna, Riyad Allehebi, Hussein Al-Matar, Mohamed Fattouh, Maarten Beekman

**Affiliations:** 1 Department of Medicine - Pulmonary Division, King Abdullah International Medical Research Center, King Saud bin Abdulaziz University for Health Sciences, Riyadh, Saudi Arabia; 2 Department of Medicine - Pulmonary Division, College of Medicine, King Abdulaziz University, Jeddah, Saudi Arabia; 3 Department of Medicine - Pulmonary Division, King Fahad Medical City, Riyadh, Saudi Arabia; 4 Department of Medicine, Imam Abdulrahman Al Faisal Hospital, Dammam, Saudi Arabia; 5 AstraZeneca GCC, Jeddah, Saudi Arabia; 6 AstraZeneca International, Amsterdam, Netherlands

**Keywords:** Eosinophilic asthma, Atopic asthma, Saudi Arabia, Disease characteristics, Severe asthma

## Abstract

**Background:**

Eosinophilia is a significant factor in asthma severity; however, the prevalence of severe eosinophilic asthma in Saudi Arabia is largely unknown. We aimed to determine the prevalence of the eosinophilic (defined in this study as ≥ 300 cells/mm^3^ in blood), atopic (atopic phenotype 1, defined in this study as > 100 IU/mL total serum IgE; atopic phenotype 2, defined in this study as > 150 IU/mL), and overlap phenotypes among patients with severe asthma in Saudi Arabia.

**Methods:**

A cross-sectional study was conducted in centers specialized in severe asthma management. Patients aged ≥ 12 years with severe asthma were enrolled. Study patients responded to the Global Initiative for Asthma 2018 assessment of asthma control questionnaire and provided study investigators with current information related to the study objectives. Additional medical record data and a blood sample for total serum IgE and complete blood count were collected.

**Results:**

A total of 101 patients were enrolled; 83% were female and the mean (standard deviation) age was 48.7 (13.2) years. Forty-five (45%) patients had the eosinophilic phenotype, 50 (50%) had atopic phenotype 1, and 25 (25%) had phenotypic overlap (eosinophilic and atopic 1). Forty-one (41%) patients had atopic phenotype 2 and 23 (23%) had phenotypic overlap (eosinophilic and atopic 2). Asthma control and oral corticosteroid use patterns were similar and there were no significant differences in number of asthma exacerbations across phenotypes.

**Conclusions:**

In Saudi Arabia, 45% of patients with severe asthma had the eosinophilic phenotype, which is most likely an underestimation as no clinical features of eosinophilia were taken into account in the definition of eosinophilia. Approximately half of them had phenotypic overlap with the atopic phenotype.

*Trial registration* NCT03931954; ClinicalTrials.gov, April 30, 2019.

**Supplementary Information:**

The online version contains supplementary material available at 10.1186/s12890-022-01856-9.

## Background

Asthma has a global prevalence of approximately 339 million and is a leading cause of mortality [1]. It affects approximately 2 million people in Saudi Arabia [2]. Although therapy is available, approximately 3–10% of asthmatic patients present with severe disease [3], which is defined as asthma that requires treatment with high-dose inhaled corticosteroids (ICS) plus a second controller to prevent it from becoming ‘uncontrolled’, or asthma that remains ‘uncontrolled’ despite treatment [4]. Severe asthma is a major unmet need and may represent up to 50% of total asthma-related healthcare costs [5, 6]. Severe asthma can be divided into several phenotypes, of which severe eosinophilic asthma is among the most studied [7]. A small study in India used cluster analysis to identify four distinct clinical phenotypes of asthma; two of them had poor attainment of maximum lung function, demonstrating that phenotype is important for understanding prognosis [8]. Biomarkers may be a useful to aid in phenotype determination. For example, a study conducted in Egypt found an association between sputum periostin and sputum eosinophilia [9]. Another study reported that patients admitted to the hospital due to asthma exacerbations who had a blood eosinophil count ≥ 300 cells/mm^3^ at admission had a > 40% reduction of risk of late readmission [10]. Eosinophils are recognized as a significant biomarker for determining disease severity; the persistent airway inflammation that occurs with eosinophils is partly responsible for the high frequency of exacerbations seen in severe asthma [11, 12]. Both phenotype and biomarkers are important to consider when identifying appropriate treatment [13, 14]. Patients with severe eosinophilic asthma utilize healthcare resources more frequently, incur higher disease management expenses, and experience a worse quality of life compared with patients with non-eosinophilic asthma phenotypes [11, 15]. Despite its impact, severe eosinophilic asthma is yet to be clearly defined. Peripheral blood eosinophil counts as high as 400 cells/mm^3^ have been linked to increased asthma exacerbations; however, patients with adult-onset asthma who have blood eosinophil counts ≥ 300 cells/mm^3^ present with a distinct phenotype of severe asthma that includes frequent exacerbations and a poor prognosis [16]. Blood eosinophil counts ≥ 200 cells/mm^3^ have been associated with asthma exacerbation, including asthma-related outpatient visits and emergency department visits [17]. Studies of anti-eosinophilic therapies indicate that patients with blood eosinophil counts ≥ 300 cells/mm^3^ can benefit from targeted treatment [18–20]. Classically, eosinophilic airway inflammation has been associated with allergic asthma [21]; however, there is evidence that eosinophilia is present in the airways of patients with severe asthma who do not have allergic disease [22] and it is suggested that a mechanism of eosinophilic asthma other than allergy may be responsible for adult-onset eosinophilic asthma as it frequently develops without allergen-dependent activation of Th2 lymphocytes [23]. Knowledge of the prevalence of eosinophilic asthma in Saudi Arabia is limited. Because management of this phenotype is complex, it is important to understand the pathophysiologic characteristics of this patient population to effectively control disease. Additionally, given the emergence of new treatment options for different phenotypes, it is important to collect local data on the prevalence of these different phenotypes to aid clinicians in selecting the right patient for the right treatment. The aim of the cross-sectional, multicenter PREPARE study was to determine the prevalence of the eosinophilic phenotype (defined in this study as ≥ 300 cells/mm^3^) among severe asthma patients in Saudi Arabia.

## Methods

### Study participants

According to the inclusion criteria, individuals were included in the study if they were ≥ 12 years of age at the time of study entry and had a diagnosis of severe asthma for at least one year, which was defined based on the Global Initiative for Asthma (GINA) 2018 guideline-suggested medications as described in Steps 4–5 (treatment with medium/high-dose ICS/long-acting beta-agonist [LABA] plus a second controller to prevent it from becoming ‘uncontrolled’, or asthma that remains ‘uncontrolled’ despite treatment) [24], i.e., the patient had to be on GINA suggested treatment for severe asthma for at least one year prior to the index visit. We note that it was not a requirement that a patient had spirometry-diagnosed asthma. In cases where spirometry data were not available, patients were diagnosed based on clinical response to trials of inhaled steroid (based on the GINA criteria for severe asthma) and through differential diagnosis. According to the exclusion criteria, individuals with a diagnosis of any chronic respiratory condition other than asthma, who had an acute or chronic condition that was considered by the study investigator to limit their ability to participate in the study, or who were currently being treated with a biologic therapy for their severe asthma were not eligible to participate in the study. Patients who met all of the inclusion criteria and none of the exclusion criteria were invited to participate in the study.

### Study design

The PREPARE study was a multicenter, cross-sectional study that included retrospective data collection to assess the prevalence of eosinophilic and atopic phenotypes among severe asthma patients. Asthma control was also studied. The study was conducted in centers that specialized in the management of severe asthma located in Saudi Arabia. Patients were identified and consecutively invited to participate in the study at their routine clinical appointment. The enrollment period was planned to be approximately 8 months or until the required number of patients had been recruited, whichever occurred first.

Early onset asthma was defined as asthma diagnosed at < 12 years of age; late onset asthma was defined as asthma diagnosed at ≥ 12 years of age [25]. Severe asthma exacerbations were defined using the criteria of the official American Thoracic Society/European Respiratory Society statement on asthma control [26] and included events that required urgent action by the patient or physician to prevent a serious outcome, such as hospitalization or death, from asthma and at least one of the following: use of systemic corticosteroids, or an increase from a stable maintenance dose, for at least 3 days or hospitalization or emergency room visit because of asthma that required systemic corticosteroids. A corticosteroid burst was defined as the use of an intravenous or oral corticosteroid for at least 3 days or the use of a single intramuscular corticosteroid dose; patients on maintenance oral corticosteroids were considered to have a corticosteroid burst if their maintenance dose was increased by at least double for at least 3 days. Chronic oral corticosteroid use was defined as continuous treatment with ≥ 5 mg OCS to control asthma for the previous 12 months. Measures used to minimize selection bias included consecutive enrollment, collection of blood at the time of study visit, exclusion of patients under biologic therapy, and the participation of research centers that are specialized in the management of patients with severe asthma.

### Data collection

Data from patients who provided written informed consent was obtained from medical records and during the study visit. Immediately after providing informed consent, patients were asked to respond to the GINA 2018 assessment of asthma control questionnaire [24] and to provide current information related to variables aligned with the study objectives (age, gender, height, body mass index, insurance status, educational level, current pharmacological treatment for asthma). Data for retrospective variables were collected from medical records and included the most recent spirometry assessment (conducted within one year of study entry and not during an asthma exacerbation) and the last 5 years of blood count history. A blood sample was collected at the study visit to assess total serum IgE and complete blood count, including eosinophils. Comorbidities considered associated with Type 2 inflammation were reported based on patients’ history or chart review, patient medication, and coding.

### Outcomes

The primary objective was to determine the prevalence of the eosinophilic asthma phenotype, defined as a blood eosinophil count ≥ 300 cells/mm^3^, among severe asthma patients. Although there is no clear definition of the eosinophilic asthma phenotype, we considered this to be appropriate as most clinical trials for anti-IL-5 and anti-IL5 receptor biological therapy used a cutoff of ≥ 300 cells/mm^3^ as a biomarker for severe eosinophilic asthma and an indication of therapy initiation [18–20]. Additionally, Zeiger et al. reported that eosinophil counts of ≥ 300 cells/mm^3^ were associated with a significantly increased risk of exacerbation (adjusted rate ratio, 1.25; 95% confidence interval [CI], 1.04–1.51) [27]. Secondary objectives included determination of the prevalence of the following: suggested atopic phenotype as defined by >100 IU/mL total serum IgE (atopic phenotype 1), atopic phenotype as defined by > 150 IU/mL total serum IgE (atopic phenotype 2), and the overlap of atopic and eosinophilic phenotypes among severe asthma patients [28]. Skin tests and radioallergosorbent (RAST) tests were not available at many of the centers included in this study. As there is no agreed upon definition of atopic asthma in the absence of skin or RAST tests, we used former criteria to define it. Patients who met the criteria for suggested atopic phenotype 1 or 2 and also had eosinophils were considered as an overlap phenotype. Additional secondary objectives were to describe the demographic and clinical characteristics (asthma control assessment [GINA], patient medical history including asthma characterization, and laboratory tests) of this patient population, as well as the patterns of corticosteroid use (chronic treatment and/or corticosteroid bursts); to determine the number of severe asthma exacerbations in the previous 12 months according to asthma phenotype (total number of severe exacerbations reported in the year prior to study entry/number of included patients); and to evaluate asthma control using the GINA 2018 assessment of asthma control [24].

### Statistical methods

Sample size was determined as follows. Schleich et al. found that 53% of patients from the Belgian Severe Asthma Registry had elevated blood eosinophil counts (> 220 cells/mm^3^) [29]. Based on this, a conservative estimate of 50% prevalence of patients having an elevated blood eosinophil count (≥ 300 cells/mm^3^) was used in the present study. For a sample size of 100 patients, the expected 95% confidence interval for an observed proportion of 50% was expected to be between 39.8 and 60.2%; therefore, a minimum sample size of 100 patients was considered adequate to provide sufficient precision in the estimation of the primary outcome measure.

Continuous variables were summarized with descriptive statistics and categorical variables were summarized with frequency counts and percentages. Analysis of variance (ANOVA) was used to analyze the mean differences among more than two groups. All statistical tests were two-sided and a *p*-value ≤ 0.05 was considered statistically significant. All statistical analyses were of an explorative and descriptive nature. No allocations were made for missing values. Data were analyzed using R statistics version 3.6.4.

## Results

### Study participants

Between May 2019 and December 2019, 101 patients were enrolled in the study from five study sites located throughout Saudi Arabia; all 101 were included in the full analysis set. Patient characteristics (demographic and lifestyle) for the total participants and according to phenotype are described in Table 1. The mean (standard deviation [SD]) age was 48.7 (13.2) years, 83% of patients were female, and 91% had never smoked. Patient characteristics were similar among the phenotype subgroups. Of note, 82% of patients in the present study were overweight (≥ 25–29.9 body mass index [BMI]; 25%) or obese (≥ 30 BMI; 57%).

**Table 1:** Patient characteristics

Demographics or lifestyle characteristic	Total participants (*N* = 101)	Eosinophilic phenotype (*n* = 45)	Atopic 1 (*n* = 50)	Overlap 1 (eosinophilic/ atopic 1) (*n* = 25)	Atopic 2 (*n* = 41)	Overlap 2 (eosinophilic/ atopic 2) (*n* = 23)
Age, years (mean ± SD)	48.7 ± 13.2	50.3 ± 13.6	47.7 ± 12.8	48.7 ± 10.5	47.3 ± 12.9	49.0 ± 10.8
Age group						
≤ 18 years	1 (1)	0 (0)	1 (2)	0 (0)	1 (2)	0 (0)
18–54 years	68 (67)	28 (62)	33 (66)	17 (68)	27 (66)	15 (65)
≥ 55 years	32 (32)	17 (38)	16 (32)	8 (32)	13 (32)	8 (35)
Gender						
Female	84 (83)	35 (78)	39 (78)	18 (72)	30 (73)	16 (70)
Male	17 (17)	10 (22)	11 (22)	7 (28)	11 (27)	7 (30)
Height, cm (mean ± SD)	157.1 ± 8.8	157.0 ± 10.1	157.9 ± 10.0	158.7 ± 10.7	158.4 ± 10.2	158.2 ± 10.8
Weight, kg (mean ± SD)	79.1 ± 19.2	77.6 ± 21.4	79.8 ± 19.4	82.0 ± 23.6	78.1 ± 19.4	79.5 ± 22.5
BMI, kg/m^2^ (mean ± SD)	32.0 ± 7.2	31.9 ± 6.9	32.2 ± 7.9	31.0 ± 7.0	31.4 ± 7.8	31.9 ± 6.9
BMI group						
< 18.5	1 (1)	1 (2)	0 (0)	0 (0)	0 (0)	0 (0)
18.5–24.9	17 (17)	9 (20)	9 (18)	5 (20)	9 (22)	5 (22)
≥ 25–29.9	25 (25)	15 (33)	15 (30)	8 (32)	14 (34)	8 (35)
≥ 30	58 (57)	20 (44)	26 (52)	12 (48)	18 (44)	10 (43)
Smoking history						
Former smoker	9 (9)	6 (13)	5 (10)	4 (16)	4 (10)	3 (13)
Never smoker	92 (91)	39 (87)	45 (90)	21 (84)	37 (90)	20 (87)

Data are shown as n (%) unless otherwise indicated

*BMI* body mass index, *SD *standard deviation

### Outcomes

The prevalence of the eosinophilic phenotype (defined in this study as ≥ 300 cells/mm^3^) was 45% (n = 45) among patients with severe asthma. The number of patients with blood eosinophil counts of 150–299 or < 150 cells/mm^3^ was 21% and 35%, respectively. As for the definition of eosinophilic phenotype, a definitive diagnosis requires evidence of eosinophilia in bronchial biopsies or induced sputum, which is challenging in clinical practice [30]. Although patients can be identified in the clinic can be identified by typical symptoms of this phenotype, such as few allergies, dyspnea on exertion, typical lung abnormalities (fixed airflow obstruction, reduced forced vital capacity, increased residual volume), typical comorbidities (nasal polyposis), and response to systemic corticosteroids [30], no clinical features of eosinophilia were included in the definition. For these reasons, the authors expect that the prevalence of 45% is most likely underestimated. The prevalence of atopic phenotype 1 was 50% (n = 50); overlap 1 (eosinophilic and atopic 1) occurred in 25% (n = 25) of patients. Atopic phenotype 2 was reported for 41% (n = 41) of patients and overlap 2 (eosinophilic and atopic 2) was reported for 23% (n = 23) of patients (Table 2). Only 18% of patients had positive specific IgE values which is evidence of sensitization to perennial allergens.

**Table 2:** Distribution of asthma phenotypes among the study participants

	Total participants (N = 101)
	N (%)
Phenotype	
Eosinophilic phenotype (≥ 300 cells/mm^3^)	45 (45)
Atopic phenotype 1 (total serum IgE > 100 IU/mL)	50 (50)
Overlap (eosinophilic and atopic 1)	25 (25)
Atopic phenotype 2 (total serum IgE > 150 IU/mL)	41 (41)
Overlap (eosinophilic and atopic 2)	23 (23)
Eosinophil group	
≥ 300 cells/mm^3^	45 (45)
150–299 cells/mm^3^	21 (21)
< 150 cell/mm^3^	35 (35)

Data are shown as *n* (%)

Disease characteristics for the total population and according to phenotype are shown in Table 3.

**Table 3:** Disease characteristics

Disease characteristic	Total participants (*N *= 101)	Eosinophilic phenotype (*n *= 45)	Atopic 1 (*n *= 50)	Overlap 1 (eosinophilic/atopic 1) (*n *= 25)	Atopic 2 (*n *= 41)	Overlap 2 (eosinophilic/atopic 2) (*n *= 23)
Asthma duration, years (mean ± SD)	17.6 ± 12.1	19.4 ± 12.8	15.6 ± 11.5	16.5 ± 12.7	16.6 ± 12.1	17.2 ± 12.9
Number of severe exacerbations (mean ± SD)	2.7 ± 4.2	2.4 ± 3.4	3.2 ± 4.0	2.9 ± 3.9	3.3 ± 4.3	3.0 ± 4.0
Number of severe asthma exacerbations						
0	24 (24)	9 (20)	7 (14)	3 (12)	5 (12)	2 (9)
1	21 (21)	11 (24)	10 (20)	5 (20)	8 (20)	5 (22)
2	24 (24)	12 (27)	15 (30)	10 (40)	13 (32)	10 (43)
3	9 (9)	6 (13)	2 (4)	1 (4)	2 (5)	1 (4)
> 3	23 (23)	7 (16)	16 (32)	6 (24)	13 (32)	5 (22)
Asthma classification						
Early-onset (< 12 years old at diagnosis)	14 (14)	9 (20)	5 (10)	4 (16)	5 (12)	4 (17)
Late-onset (≥ 12 years old at diagnosis)	87 (86)	36 (80)	45 (90)	21 (84)	36 (88)	19 (83)
History of atopy						
No	83 (82)	35 (78)	38 (76)	16 (64)	30 (73)	15 (65)
Yes	18 (18)	10 (22)	12 (24)	9 (36)	11 (27)	8 (35)
Specific IgE available in medical records						
No	3 (3)	2 (4)	1 (2)	1 (4)	1 (2)	1 (4)
Yes	38 (38)	14 (31)	18 (36)	10 (40)	14 (34)	9 (39)
Negative	20/38 (53)	5/14 (36)	4/18 (22)	2/10 (20)	2/14 (14)	1/9 (11)
Positive	18 /38(47)	9/14 (64)	14/18 (78)	8/10 (80)	12/14 (86)	8/9 (89)
Missing	60 (59)	29 (64)	31 (62)	14 (56)	26 (63)	13 (57)
Comorbidities						
No	35 (35)	21 (48)	20 (40)	14 (56)	18 (44)	13 (57)
Yes	65 (65)	23 (52)	30 (60)	11 (44)	23 (56)	10 (43)
Missing	1 (1)	1 (2)	0 (0)	0 (0)	0 (0)	0 (0)

Data are shown as n (%) unless otherwise indicated

*SD* standard deviation

The mean (SD) duration of asthma was 17.6 (12.1) years and 87 (86%) patients had late-onset asthma. The majority of patients (82%) did not have a history of atopy. Prevalence of comorbidities of interest among the 65 patients (total participants) with comorbidities was as follows: rhinitis, 65%; nasal polyps, 26%; atopic dermatitis, 3%; sinusitis, 22%; and eczema, 2%. In total, 77% of patients had at least one asthma exacerbation within the previous 12 months, and 32% had ≥ 3 exacerbations in the same time period. Among patients with the eosinophilic phenotype, 29% had ≥ 3 exacerbations in the previous 12 months. Additionally, clinical parameters for the total participants are shown in Table 4. A spirometry assessment was available for 75 (75%) patients. The study visit blood test revealed that the median (interquartile range [IQR]) of absolute eosinophils was 250.0 (110.0, 500.0) cells/mm^3^ and that the median (IQR) for total serum IgE was 99.5 (38.0, 289.0) IU/mL (Additional file 1).

**Table 4:** Clinical parameters

	Total participants (N = 101)
Spirometry assessment (most recent)	
Participants with assessment available, *n* (%)	75 (75%)
Pre-BD FVC	2.6 ± 0.8
Post-BD FVC*	2.7 ± 0.8
Pre-BD % of the predicted FVC value	83.4 ± 17.6
Post-BD % of the predicted FVC value^†^	82.9 ± 16.9
Pre-BD FEV1	2.0 ± 0.7
Post-BD FEV1^‡^	2.0 ± 0.7
Pre-BD % of the predicted FEV1 value^§^	77.9 ± 20.7
Post-BD % of the predicted FEV1 value^†^	80.8 ± 18.9
Pre-BD FEV1/FVC	76.3 ± 11.2
Post-BD FEV1/FVC^‡^	76.7 ± 10.9
Blood test (at study visit)	
WBC (cells/mm^3^)	7300.0 (6100.0, 8910.0)
Eosinophils (%)	3.3 (1.7, 6.3)
Absolute eosinophils (cells/mm^3^)	250.0 (110.0, 500.0)
Total serum IgE (IU/mL)	99.5 (38.0, 289.0)

Data are shown as mean ± SD or median (IQR) unless otherwise indicated

BD = bronchodilator; FEV = forced expiratory volume; FVC = forced vital capacity; GINA = Global Initiative for Asthma; SD = standard deviation, IQR = interquartile range (25th percentile, 75th percentile); WBC = white blood cell

*23/75 (31%) patients had missing values

^†^34/75 (45%) patients had missing values

^‡^24/75 (32%) patients had missing values

^§^1/75 (1%) patient had a missing value

**Fig. 1:**
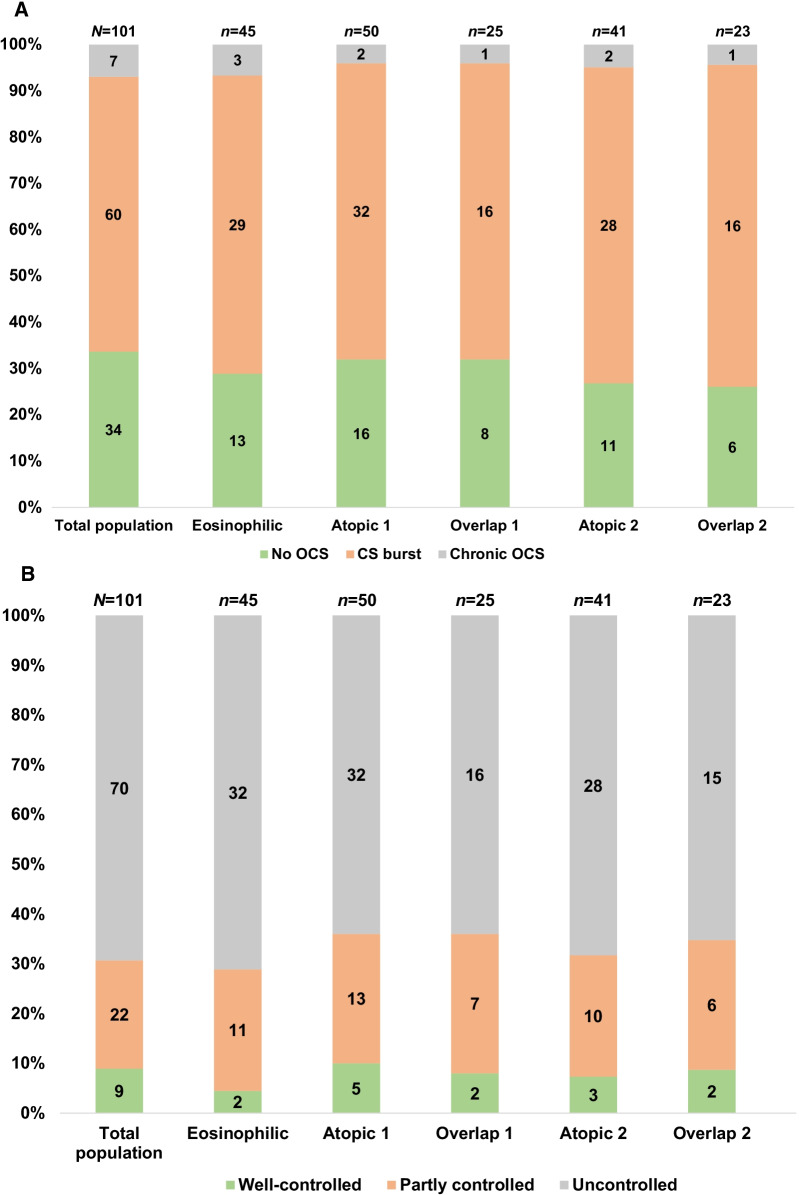
**A** Pattern of oral corticosteroid use across phenotypes. **B** Asthma control among study subjects

Asthma treatments in the 12 months prior to study enrollment for the total participants are shown in Table 5. Almost all participants (n = 99, 98%) received treatment with a fixed dose combination of ICS/LABA. Most participants were not on chronic oral corticosteroids (n = 94, 93%); prednisone was the only drug used in patients who were on chronic oral corticosteroid treatment. Sixty (60%) patients received corticosteroid burst treatment, with prednisone being the treatment received by most (n = 55, 93%). Thirty-seven (45%) patients received three or more corticosteroid burst treatments. Figure 1A shows that the pattern of oral corticosteroid use was similar across phenotypes.

**Table 5:** Asthma treatments in the 12 months prior to study enrollment

	Total participants (N = 101)
Chronic OCS, no/yes	94 (93)/7 (7)
Chronic OCS treatment ( N = 7)*	
Prednisone	7 (100%)
Total daily dose, mg	10.7 ± 6.7
Total exposure (12 months), mg	3679.3 ± 2703.1
CS burst treatment^†^, no/yes	40 (40%)/60 (60%)
Active substance (N = 59)^*,†^	
Hydrocortisone	1 (2%)
Methylprednisolone	3 (5%)
Prednisone	55 (93%)
Total number (12 months) (N = 60)*	
1	13 (22%)
2	20 (33%)
3	10 (17%)
> 3	17 (28%)
ICS/LABA (fixed dose combination), no/yes	2 (2%)/99 (98%)
Active substance ( N = 99)*	
Beclomethasone/formoterol	1 (1%)
Budesonide/formoterol	52 (53%)
Fluticasone/salmeterol	44 (44%)
Fluticasone/vilanterol	2 (2%)
Total daily dose (N = 99)*^,‡^	
Low dose	2 (2%)
Medium dose	50 (51%)
High dose	47 (47%)

Data are shown as *n* (%) or mean ± SD

*CS *corticosteroid, *ICS* inhaled corticosteroid, *LABA *long-acting beta-agonist, *OCS *oral corticosteroid, *SD *standard deviation

*Treatment information is given for the patients who answered Yes to the corresponding question about treatment

^†^Information was missing for one patient

^‡^Low, medium, and high dose was defined according to the Global Asthma Report 2018 [1]

At the time of the study visit, 69% (n = 70) of patients had uncontrolled asthma, 22% (n = 22) had partially controlled asthma, and 9% (n = 9) had well-controlled asthma (Fig. 1B). The distribution of asthma control level was similar across the different phenotypes.

**Fig. 2:**
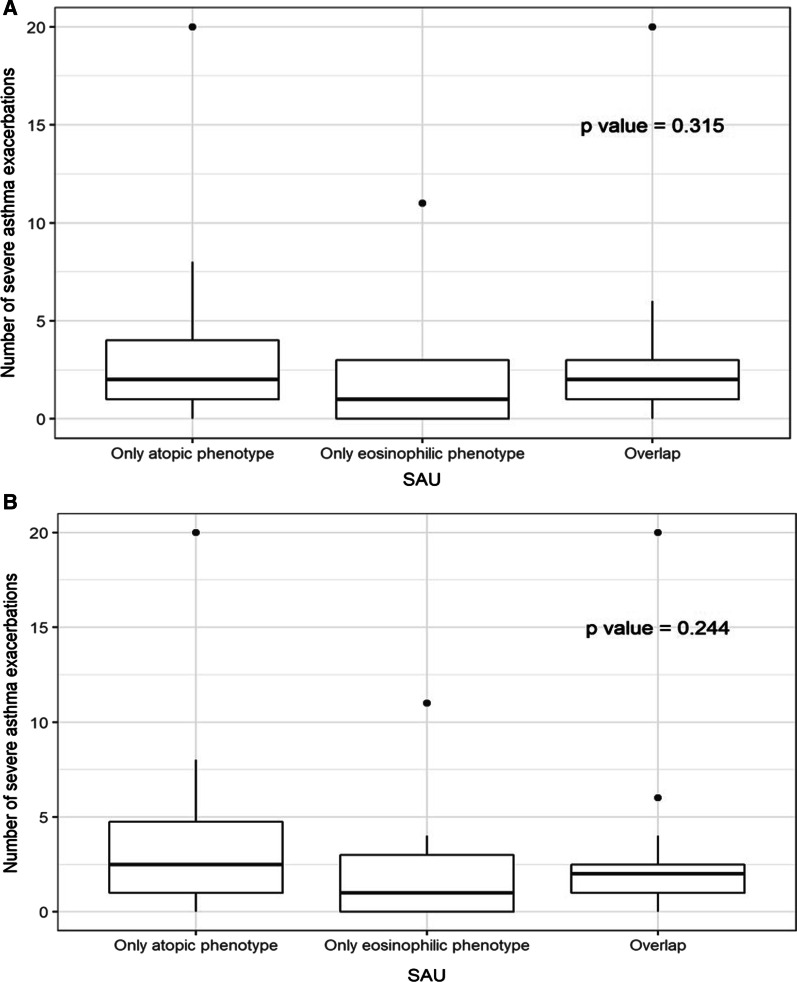
**A** ANOVA test of differences in number of severe exacerbations across phenotype in SAU (atopic phenotype as total serum IgE > 100 IU/mL). **B** ANOVA test of differences in number of severe exacerbations across phenotype in SAU (atopic phenotype as total serum IgE > 150 IU/mL)

There were no significant differences in the number of severe asthma exacerbations (in the year prior to study enrollment) among patients with only atopic 1, only eosinophilic, or overlap 1 asthma phenotypes (*P* = 0.315; Fig. 2A) or among patients with only atopic 2, only eosinophilic, and overlap 2 asthma phenotypes (*P* = 0.244; Fig. 2B).

## Discussion

This cross-sectional study found that the eosinophilic phenotype occurred in 45% of patients with severe asthma in Saudi Arabia. The atopic phenotype was also quite prevalent in this patient population (atopic phenotype 1, 50%; atopic phenotype 2, 41%). Given that only 18% of patients had positive specific IgE values, which is evidence of sensitization to perennial allergens, the prevalence of the atopic phenotypes 1 and 2 are most likely overestimated. Approximately half of patients with the eosinophilic phenotype had phenotypic overlap with the suggested atopic phenotype; overlap patients accounted for approximately one-quarter of patients overall. Most patients (70%) had uncontrolled asthma and 60% received corticosteroid burst treatment in the past 12 months of which 45% received three or more bursts. The pattern of corticosteroid use and level of asthma control were similar across phenotypes. There were no significant differences in the number of asthma exacerbations in the 12 months prior to the study among phenotypes. It should be noted that the large amount of overlap within the phenotypic groups may have accounted for the observation that characteristics and outcomes were similar among phenotypes.

The prevalence of the eosinophilic asthma phenotype among severe asthma patients reported in the present study is similar to that reported in other geographic regions (Australia, 44%; Japan, 34%; Netherlands, 44%) [31–33]. In the present study, the definition of eosinophilic phenotype was only based on a blood eosinophil count of ≥ 300 cells/mm^3^. This is most likely an underestimation of the real prevalence as clinical features of eosinophilia, like nasal polyps, adult-onset asthma, and chronic OCS use were not taken into account [34]. The prevalence of the suggested atopic phenotypes 1 and 2 was defined by cut off values of serum IgE, but to really define atopy, evidence of sensitization to perennial allergens is needed as defined by positive specific IgE values. In 60% of the cases, no specific IgE values were available in the medical records and of the ones that were available, less than half were positive. Therefore, we think that the percentages of the suggested atopic phenotypes 1 and 2 are most likely overestimated. The extent of phenotypic overlap is largely unknown. The present study reports 23–25% phenotypic overlap between eosinophilic and atopic phenotypes, depending on the cutoff used to define the atopic phenotype. Again, this study was limited in the ability to truly categorize patients as atopic. We found that only 9% of patients in our population had well-controlled asthma. A better understanding of the occurrence and classification of the different phenotypes of severe asthma may lead to better treatment strategies. Patients with eosinophilic asthma are eligible for eosinophil-targeting biologics, such as anti-IL-5 or anti-IL-5 receptor antibody therapy [24].

The management of severe asthma is complex and requires a thorough and up to date understanding of the pathophysiologic characteristics of this patient population to facilitate effective therapeutic decision-making. The high incidence of overweight or obese participants observed in the present study warrants a call to action among patients with severe asthma in Saudi Arabia, as an increased BMI is associated with an increased risk of asthma exacerbations [35], and weight loss has been shown to improve asthma-related quality of life, asthma control, and lung function in obese patients with asthma [36]. Interestingly, the recently published Spanish Asthma guidelines do not consider the severe asthma phenotype linked with obesity, which is a change from previous years [37]. The present study provides new information regarding the incidence of eosinophilic, atopic, and overlapping phenotypes among patients with severe asthma, as well as the disease characteristics for each of these populations. This information will help inform physicians treating this patient population. As an example, the present study reported a high percentage of participants with the eosinophilic phenotype who experienced ≥ 3 asthma exacerbations over a period of 12 months. Anti-IL-5 or anti-IL-5 receptor biologic therapy has been shown to reduce the number of exacerbations in patients with eosinophilic asthma and would therefore likely benefit this patient population [38]. While biologic therapy is beneficial for patients with eosinophilic asthma, a recent study conducted in Brazil found that many patients with severe asthma were not eligible for approved biologics and that those who were eligible may not be able to access them [39]. Additionally, it has been reported that even patients who have well-controlled severe asthma may experience low asthma-related quality of life [40]. This indicates a need for continued efforts to develop treatment alternatives for patients who are not eligible to receive biological therapies or who experience a low quality of life despite having well-controlled asthma. Future studies to understand optimal treatment management based on phenotype are warranted.

This study had several limitations. This study utilized consecutive enrollment, which may have resulted in low internal validity and limited generalizability given the non-probabilistic sampling technique. The cross-sectional study design does not allow for the establishment of cause-effect relationships and the partial reliance on retrospectively collected data may have resulted in some incidences of incomplete, poorly recorded, or absent data. The definitions of eosinophilic and atopic phenotypes were only based on blood eosinophil and serum IgE levels, respectively, and did not include any clinical features of eosinophilic asthma nor were data on sensitization of perennial allergens included. Comorbidities of sleep apnea, gastroesophageal reflux, anxiety, and depression, which are relevant for patients with asthma [41, 42], were not reported for any of the patients included in the study; however, given the observational nature of the study, it is possible that these comorbidities were present in some patients but that this information was not included in the patient’s history. This study did not include patients who were receiving biologic therapy for asthma treatment; because biologics are approved for use in patients with severe asthma, it is possible that a subset of patients with more severe disease were excluded from the study. Finally, this study was partially reliant on patient reported data, which may be subject to recall bias and non-response bias.

## Conclusion

Overall, this study reported a prevalence of 45% for the eosinophilic phenotype (≥ 300 cells/mm^3^) among patients with severe asthma in Saudi Arabia, which is most likely an underestimation of the real prevalence. Additionally, 41–50% of patients with severe asthma had the atopic phenotype (depending on whether the cutoff was > 150 IU/mL or >  IU/mL total serum IgE, respectively), which is most likely an overestimation of the real prevalence given the limitations in defining the atopic phenotype. In total, 23–25% of patients had phenotypic overlap between eosinophilic and atopic phenotypes, which accounted for approximately half of the patients with each phenotype. Patient demographic and clinical characteristics were similar across the different phenotypes, as were corticosteroid use patterns, level of asthma control, and incidence of exacerbations. Considering the impact of severe asthma on healthcare systems and the current lack of data around available biomarkers to guide precision therapy, it is important to have prospective local registries to better capture patient data, which may help to guide treatment. The results of the present study serve to inform treating physicians of the importance of phenotyping to select the right biologic treatment.

## Supplementary Information

**Additional file 1.** Statistical Analysis Report.

## Data Availability

The datasets used during the current study are available from the corresponding author upon request.

## References

[1] Global Asthma Network [homepage on the Internet]. The global asthma report. 2018. http://www.globalasthmareport.org/Global%20Asthma%20Report%202018.pdf. Accessed 12 Sept 2020.

[2] Mohamed Hussain S, Ayesha Farhana S, Mohammed Alnasser S (2018). Time trends and regional variation in prevalence of asthma and associated factors in Saudi Arabia: a systematic review and meta-analysis. Biomed Res Int.

[3] Israel E, Reddel HK (2017). Severe and difficult-to-treat asthma in adults. N Engl J Med.

[4] Chung KF, Wenzel SE, Brozek JL, Bush A, Castro M, Sterk PJ (2014). International ERS/ATS guidelines on definition, evaluation and treatment of severe asthma. Eur Respir J.

[5] Chastek B, Korrer S, Nagar SP, Albers F, Yancey S, Ortega H (2016). Economic burden of illness among patients with severe asthma in a managed care setting. J Manag Care Spec Pharm.

[6] Nunes C, Pereira AM, Morais-Almeida M (2017). Asthma costs and social impact. Asthma Res Pract.

[7] Buhl R, Humbert M, Bjermer L, Chanez P, Heaney LG, Pavord I (2017). Severe eosinophilic asthma: a roadmap to consensus. Eur Respir J.

[8] Bhargava S, Holla AD, Jayaraj BS, Praveena AS, Ravi S, Khurana S (2021). Distinct asthma phenotypes with low maximal attainment of lung function on cluster analysis. J Asthma.

[9] Refaat MM, El Sayed E, El-Fattah WA, Elbanna AH, El Sayed HM (2021). Relationship between sputum periostin level and inflammatory asthma phenotypes in Egyptian patients. J Asthma.

[10] Gonzalez-Barcala FJ, San-Jose ME, Nieto-Fontarigo JJ, Carreira JM, Calvo-Alvarez U, Cruz MJ (2018). Association between blood eosinophil count with asthma hospital readmissions. Eur J Intern Med.

[11] Schleich FN, Louis R (2014). Importance of concomitant local and systemic eosinophilia in uncontrolled asthma. Eur Respir J.

[12] Green RH, Brightling CE, McKenna S, Hargadon B, Parker D, Bradding P (2002). Asthma exacerbations and sputum eosinophil counts: a randomized controlled trial. Lancet.

[13] Casan CP, Gonzalez CM (2020). Biologics in the treatment of asthma. Arch Bronconeumol (Engl Ed).

[14] Arismendi E, Valles CP (2020). Current role of biomarkers in severe uncontrolled asthma. Arch Bronconeumol (Engl Ed).

[15] Husereau D, Goodfield J, Leigh R, Borrelli R, Cloutier M, Gendron A, Severe (2018). eosinophilic asthma in primary care in Canada: a longitudinal study of the clinical burden and economic impact based on linked electronic medical record data. Allergy Asthma Clin Immunol.

[16] Price DB, Rigazio A, Campbell JD, Bleecker ER, Corrigan CJ, Thomas M (2015). Blood eosinophil count and prospective annual asthma disease burden: a UK cohort study. Lancet Resp Med.

[17] Mallah N, Rodriguez-Segade S, Gonzalez-Barcala FJ, Takkouche B (2021). Blood eosinophil count as predictor of asthma exacerbation. A meta-analysis. Pediatr Allergy Immunol.

[18] Ortega HG, Liu MC, Pavord ID, Brusselle GG, FitzGerald MJ, Chetta A (2014). Mepolizumab treatment in patients with severe eosinophilic asthma. N Engl J Med.

[19] Corren J, Weinstein S, Janka L, Zangrilli J, Garin M (2016). Phase 3 study of reslizumab in patients with poorly controlled asthma: effects across a broad range of eosinophil counts. Chest.

[20] Castro M, Wenzel SE, Bleecker ER, Pizzichini E, Kuna P, Busse WW (2014). Benralizumab, an anti-interleukin 5 receptor alpha monoclonal antibody, versus placebo for uncontrolled eosinophilic asthma: a phase 2b randomised dose-ranging study. Lancet Resp Med.

[21] Possa SS, Leick EA, Prado CM, Martins MA, Tibério IFLC (2013). Eosinophilic inflammation in allergic asthma. Front Pharmacol.

[22] Walford HH, Doherty TA (2014). Diagnosis and management of eosinophilic asthma: a US perspective. J Asthma Allergy.

[23] De Groot JC, ten Brinke A, Bel EHD (2015). Management of the patient with eosinophilic asthma: a new era begins. ERJ Open Res.

[24] Global Initiative for Asthma (GINA) [homepage on the Internet]. Global strategy for asthma management and prevention (Updated 2018). https://ginasthma.org/wp-content/uploads/2019/01/2018-GINA.pdf. Accessed 13 Sept 2020.

[25] Miranda C, Busacker A, Balzar S, Trudeau J, Wenzel SE (2004). Distinguishing severe asthma phenotypes: role of age at onset and eosinophilic inflammation. J Allergy Clin Immunol.

[26] Reddel HK, Taylor DR, Bateman ED, Boulet LP, Boushey HA, Busse WW (2009). An official American Thoracic Society/European Respiratory Society statement: asthma control and exacerbations: standardizing endpoints for clinical asthma trials and clinical practice. Am J Respir Crit Care Med.

[27] Zeiger RS, Schatz M, Li Q, Chen W, Khatry DB, Gossage D (2014). High blood eosinophil count is a risk factor for future asthma exacerbations in adult persistent asthma. J Allergy Clin Immunol Pract.

[28] Tran TN, Zeiger RS, Peters SP, Colice G, Newbold P, Goldman M (2016). Overlap of atopic, eosinophilic, and TH2-high asthma phenotypes in a general population with current asthma. Ann Allergy Asthma Immunol.

[29] Schleich F, Brusselle G, Louis R, Vandenplas O, Michils A, Pilette C (2014). Heterogeneity of phenotypes in severe asthmatics. The Belgian Severe Asthma Registry (BSAR). Respir Med.

[30] de Groot JC, ten Brinke A, Bel EHD (2015). Management of the patient with eosinophilic asthma: a new era begins. ERJ Open Res.

[31] Hiles SA, Gibson PG, McDonald VM (2021). Disease burden of eosinophilic airway disease: comparing severe asthma, COPD and asthma-COPD overlap. Respirology.

[32] Nagasaki T, Sato K, Kume N, Oguma T, Sunadome H, Ito I (2019). The prevalence and disease burden of severe eosinophilic asthma in Japan. J Asthma.

[33] van Veen IH, Ten Brinke A, Gauw SA, Sterk PJ, Rabe KF, Bel EH (2009). Consistency of sputum eosinophilia in difficult-to-treat asthma: a 5-year follow-up study. J Allergy Clin Immunol.

[34] Jackson D, Busby J, Heaney LG, Menzies-Gow A, Pfeffer P, Perez-de-Llano L, et al. A global survey of blood eosinophil distribution in severe asthma patients: data from the International Severe Asthma Registry (ISAR). InC21. Advances in adult and pediatric asthma phenotyping and endotyping. American Thoracic Society; 2020. p. A4522-A4522

[35] Schatz M, Zeiger RS, Zhang F, Chen W, Yang SJ, Camargo CA (2013). Jr. Overweight/obesity and risk of seasonal asthma exacerbations. J Allergy Clin Immunol Pract.

[36] Peters U, Dixon AE, Forno E (2018). Obesity and asthma. J Allergy Clin Immunol.

[37] Plaza V, Blanco M, Garcia G, Korta J, Molina J, Quirce S (2021). Highlights of the Spanish Asthma Guidelines (GEMA), version 5.0. Arch Bronconeumol (Engl Ed).

[38] Menzella F, Ruggiero P, Ghidoni G, Fontana M, Bagnasco D, Livrieri F (2020). Anti-IL5 therapies for severe eosinophilic asthma: literature review and practical insights. J Asthma Allergy.

[39] Marques ML, Viana KP, Dos Santos FM, Saturnino LTM, Kormann ML, Lazaridis E (2021). Severe asthma and eligibility for biologics in a Brazilian cohort. J Asthma.

[40] Varsano S, Israeli L, Shitrit D (2021). "Severe-controlled” asthma 4 years later: is it still controlled?. J Asthma.

[41] Kaplan A, Szefler SJ, Halpin DMG (2020). Impact of comorbid conditions on asthmatic adults and children. NPJ Prim Care Respir Med.

[42] Mallah N, Turner JM, Gonzalez-Barcala FJ, Takkouche B (2022). Gastroesophageal reflux disease and asthma exacerbation: a systematic review and meta-analysis. Pediatr Allergy Immunol.

