# Supplementary material for: Prevalence of eosinophilic, atopic, and overlap phenotypes among patients with severe asthma in Saudi Arabia: a cross-sectional study
Source: BMC Pulm Med. 2022 Feb 17;22:67. doi: 10.1186/s12890-022-01856-9 (PMC8855582; doi:10.1186/s12890-022-01856-9)
Supplement: Supplementary file 1 — Additional file 1. Statistical Analysis Report. [file 12890_2022_1856_MOESM1_ESM.docx]

PREPARE Analysis Results for SAU

Contents

[1 SUBJECT POPULATION 2](#_Toc71463439)

[Table 1.1 Patient Disposition (Saudi patients) 2](#_Toc71463440)

[Table 1.2 Patient Recruitment by Site (All Enrolled) 3](#_Toc71463441)

[2 DEMOGRAPHIC AND LIFESTYLE CHARACTERIZATION 4](#_Toc71463442)

[Table 2.1 Demographic and Lifestyle Characteristics 4](#_Toc71463443)

[3 DISEASE CHARACTERIZATION 6](#_Toc71463444)

[Table 3.1 Disease Characteristics 6](#_Toc71463445)

[Table 3.2 Asthma Treatments in the Last 12 Months 8](#_Toc71463446)

[Table 3.3 Spirometry Assessment 10](#_Toc71463447)

[Table 3.4 Blood Test at Visit (in visit window) 13](#_Toc71463448)

[Table 3.5 Phenotype 14](#_Toc71463449)

[4 ASTHMA SYMPTOM CONTROL 15](#_Toc71463450)

[Table 4.1 Asthma Symptom Control 15](#_Toc71463451)

[5 EOSINOPHILIC ASTHMA PATIENTS SUB-GROUP ANALYSIS 16](#_Toc71463452)

[Table 5.1 Demographic and Lifestyle Characteristics by Phenotype in SAU 16](#_Toc71463453)

[Table 5.2 Disease Characteristics by Phenotype in SAU 18](#_Toc71463454)

[Table 5.3 Pattern of CS Use by Phenotype in SAU 20](#_Toc71463455)

[Table 5.4 Chi-square Test of Association between Asthma Control Levels and Phenotype in SAU 21](#_Toc71463456)

[Chi-square Test of Association between Asthma Control Levels and Phenotype in SAU (atopic phenotype as total serum IgE > 100 IU/ml) 22](#_Toc71463457)

[Chi-square Test of Association between Asthma Control Levels and Phenotype in SAU (atopic phenotype as total serum IgE > 150 IU/ml) 23](#_Toc71463458)

[Figure 5.6 ANOVA Test of Differences in Number of Severe Exacerbations across Phenotype in SAU (atopic phenotype as total serum IgE > 100 IU/ml) 25](#_Toc71463459)

[ANOVA Test of Differences in Number of Severe Exacerbations across Phenotype in SAU (atopic phenotype as total serum IgE > 150 IU/ml) 25](#_Toc71463460)

# 1 SUBJECT POPULATION

## Table 1.1 Patient Disposition (Saudi patients)

|  | SAU |
| --- | --- |
| Patients enrolled^[a]^ | 101 |
| Full Analysis Set^[b]^ | 101 (100) |

[a] Informed consent received.

[b] Percentages are calculated from number of patients who are enrolled.

No subjects excluded from the analysis.

## Table 1.2 Patient Recruitment by Site (All Enrolled)

|  | Overall |
| --- | --- |
| **SAU** | 101 |
| **Location** |  |
| SAU-01 | 30 (29.7) |
| SAU-02 | 25 (24.8) |
| SAU-04 | 11 (10.9) |
| SAU-06 | 18 (17.8) |
| SAU-07 | 17 (16.8) |

For category variables, n (%) was used for statistics.

# 2 DEMOGRAPHIC AND LIFESTYLE CHARACTERIZATION

## Table 2.1 Demographic and Lifestyle Characteristics

| Demographic and Lifestyle Characteristics | SAU (N = 101) | |
| --- | --- | --- |
| **Age** |  |  |
| minimum | 17.1 |  |
| median (IQR) | 49.0 (38.0, 57.4) |  |
| mean (sd) | 48.7 ± 13.2 |  |
| maximum | 77.8 |  |
| **Age group** |  |  |
| <=18 | 1 (1) |  |
| 18-54 | 68 (67) |  |
| >=55 | 32 (32) |  |
| **Gender** |  |  |
| Female | 84 (83) |  |
| Male | 17 (17) |  |
| **Height (cm)** |  |  |
| minimum | 120.0 |  |
| median (IQR) | 156.0 (151.0, 163.0) |  |
| mean (sd) | 157.1 ± 8.8 |  |
| maximum | 175.0 |  |
| **Weight (kg)** |  |  |
| minimum | 30.0 |  |
| median (IQR) | 80.0 (67.1, 88.9) |  |
| mean (sd) | 79.1 ± 19.2 |  |
| maximum | 150.0 |  |
| **BMI (kg/m^2^)** |  |  |
| minimum | 15.9 |  |
| median (IQR) | 31.7 (26.2, 36.8) |  |
| mean (sd) | 32.0 ± 7.2 |  |
| maximum | 58.6 |  |
| **BMI group** |  |  |
| <18.5 | 1 (1) |  |
| 18.5-24.9 | 17 (17) |  |
| ≥25-29.9 | 25 (25) |  |
| ≥30 | 58 (57) |  |
| **Educational level** |  |  |
| Elementary school | 12 (12) |  |
| Secondary school | 12 (12) |  |
| High-school | 10 (10) |  |
| University and post-university education | 27 (27) |  |
| Unknown | 40 (40) |  |
| **Insurance status** |  |  |
| Fully Reimbursed | 69 (68) |  |
| Partially Reimbursed | 7 (7) |  |
| Not reimbursed | 24 (24) |  |
| Unknown | 1 (1) |  |
| **Smoking status history** |  |  |
| Active smoker | 0 (0) |  |
| Former smoker | 9 (9) |  |
| Never smoker | 92 (91) |  |

SD=Standard deviation; IQR=Interquartile range (25th Percentile to 75th Percentile);
for category variables, n (%) was used for statistics.

# 3 DISEASE CHARACTERIZATION

## Table 3.1 Disease Characteristics

| Disease Characteristics | SAU (N = 101) |
| --- | --- |
| **Asthma duration, year** |  |
| minimum | 1.0 |
| median (IQR) | 15.9 (8.3, 24.7) |
| mean (sd) | 17.6 ± 12.1 |
| maximum | 57.8 |
| **Number of severe asthma exacerbations in the last year** |  |
| minimum | 0.0 |
| median (IQR) | 2.0 (1.0, 3.0) |
| mean (sd) | 2.7 ± 4.2 |
| maximum | 30.0 |
| **Number of severe asthma exacerbations** |  |
| 0 | 24 (24) |
| 1 | 21 (21) |
| 2 | 24 (24) |
| 3 | 9 (9) |
| >3 | 23 (23) |
| **Asthma classification** |  |
| Early-onset asthma | 14 (14) |
| Late-onset asthma | 87 (86) |
| **History of atopy** |  |
| NO | 83 (82) |
| YES | 18 (18) |
| Missing | 0/101 (0) |
| **Skin prick test history** |  |
| NO | 39 (98) |
| YES | 1 (2) |
| Missing | 61/101 (60) |
| **If yes skin prick test** |  |
| Negative | 0 (0) |
| Positive | 1 (100) |
| **Aeroallergens positive** |  |
| N/A | 1 (100) |
| No | 0 (0) |
| Yes | 0 (0) |
| Missing | 100/101 (99) |
| **Specific IgE available in the medical records** |  |
| NO | 3 (7) |
| YES | 38 (93) |
| Missing | 60/101 (59) |
| **If yes specific IgE** |  |
| Negative | 20 (53) |
| Positive | 18 (47) |
| **Comorbidities** |  |
| NO | 35 (35) |
| YES | 65 (65) |
| Missing | 1/101 (1) |
| **Co-morbidity type*** | 65 |
| Rhinitis | 42 (64.6) |
| Nasal polyps | 17 (26.2) |
| Atopic dermatitis | 2 (3.1) |
| Asthma associated with NSAIDs | 0 (0.0) |
| Sinusitis | 14 (21.5) |
| Aspergillosis | 0 (0.0) |
| Eczema | 1 (1.5) |
| Urticaria | 0 (0.0) |

SD=Standard deviation; IQR=Interquartile range (25th Percentile to 75th Percentile);
for category variables, n (%) was used for statistics.

* Co-morbidity type: we only list interested co-morbidities, and other types of co-morbidity were not shown in the table. Percentages are calculated based on number of patients who had comorbidities.

## Table 3.2 Asthma Treatments in the Last 12 Months

| Asthma Treatments in the Last  12 Months | SAU (N = 101) |
| --- | --- |
| **Chronic OCS** |  |
| No | 94 (93) |
| Yes | 7 (7) |
| **Chronic OCS: Active substances*** |  |
| Deflazacort | 0 (0) |
| Dexamethasone | 0 (0) |
| Methyl-prednisolone | 0 (0) |
| Prednisolone | 0 (0) |
| Prednisone | 7 (100) |
| **Chronic OCS: TOTAL DAILY DOSE** |  |
| Minimum | 5.0 |
| median (IQR) | 10.0 (7.5, 10.0) |
| mean (sd) | 10.7 ± 6.7 |
| Maximum | 25.0 |
| **Chronic OCS: TOTAL EXPOSURE OVER 12 MONTH** |  |
| Minimum | 405.0 |
| median (IQR) | 3,600.0 (2,712.5, 3,600.0) |
| mean (sd) | 3,679.3 ± 2,703.1 |
| Maximum | 9,125.0 |
| Missing | 0/7 (0) |
| **CS burst treatment/short course** |  |
| No | 40 (40) |
| Yes | 60 (60) |
| Missing | 1/101 (1) |
| **CS burst treatment/short course: Active substances*** |  |
| Betametasone | 0 (0) |
| Deflazacort | 0 (0) |
| Dexamethasone | 0 (0) |
| Dexamethasone/Meprednisone | 0 (0) |
| Hydrocortisone | 1 (2) |
| Meprednisone | 0 (0) |
| Methyl-prednisolone | 3 (5) |
| Prednisolone | 0 (0) |
| Prednisone | 55 (93) |
| Missing | 1/60 (2) |
| **TOTAL NUMBER OF BURSTS/COURSES Corticosteroids OVER 12 MONTHS*** |  |
| 1 | 13 (22) |
| 2 | 20 (33) |
| 3 | 10 (17) |
| >3 | 17 (28) |
| Missing | 0/60 (0) |
| **ICS/LABA (fixed dose combination)** |  |
| NO | 2 (2) |
| YES | 99 (98) |
| **ICS/LABA: Active substances*** |  |
| Beclomethasone/Formoterol | 1 (1) |
| Budesonida | 0 (0) |
| Budesonide/Formoterol | 52 (53) |
| Fluticasone/Salmeterol | 44 (44) |
| Fluticasone/ Vilanterol | 2 (2) |
| Fluticasone/Formoterol | 0 (0) |
| Fluticasone/Umeclidinium/Vilanterol | 0 (0) |
| Mometasone/Formoterol | 0 (0) |
| **ICS/LABA: TOTAL DAILY ICS DOSE*** |  |
| Low dose | 2 (2) |
| Medium dose | 50 (51) |
| High dose | 47 (47) |
| **Other maintenance therapies** |  |
| NO | 12(12) |
| YES | 89(88) |
| **Other drug class*** |  |
| LTRA | 58(65) |
| SABA | 31(35) |
| LAMA | 36(40) |
| ICS | 2(2) |
| LABA | 0(0) |
| SAMA | 1(1) |
| Antihistamine | 0(0) |
| Xanthine | 5(6) |
| SABA/SAMA | 0(0) |
| Nasal steroid | 3(3) |
| Anti IgE Antibody | 0(0) |
| **Other drug dosage form*** |  |
| Inhalation | 59(66) |
| Tablet | 58(65) |
| Nasal | 3(3) |
| Capsulas | 0(0) |
| Injection | 0(0) |

CS=Corticosteroids; ICS=Inhaled Corticosteroid; OCS=Oral Corticosteroid; LTRA: Leukotriene Receptor Antagonists; LABA=Long Acting Beta2 Agonists;

SABA: Short Acting Beta2 Agonists; SAMA: Short Acting Muscarinic Antagonists; LAMA: Long Acting Muscarinic Antagonists;

SD=Standard deviation; IQR=Interquartile range (25th Percentile to 75th Percentile).
For category variables, n (%) was used for statistics.

* Percentages are calculated from number of patients who answered “YES” to the corresponding question about treatment.

## Table 3.3 Spirometry Assessment

| Spirometry Assessment | SAU (N = 101) |
| --- | --- |
| MOST RECENT SPIROMETRY ASSESSMENT available | 75 |
| **Pre-bronchodilator FVC** |  |
| Minimum | 0.9 |
| median (IQR) | 2.5 (2.0, 3.1) |
| mean (sd) | 2.6 ± 0.8 |
| Maximum | 4.6 |
| Missing | 0/75 (0) |
| **Post-bronchodilator FVC** |  |
| Minimum | 1.1 |
| median (IQR) | 2.6 (2.0, 3.2) |
| mean (sd) | 2.7 ± 0.8 |
| Maximum | 4.3 |
| Missing | 23/75 (31) |
| **Pre-BD % of the predicted FVC value** |  |
| Minimum | 33.0 |
| median (IQR) | 84.5 (72.0, 96.0) |
| mean (sd) | 83.4 ± 17.6 |
| Maximum | 122.5 |
| Missing | 0/75 (0) |
| **Post-BD % of the predicted FVC value** |  |
| Minimum | 45.0 |
| median (IQR) | 83.0 (70.0, 94.4) |
| mean (sd) | 82.9 ± 16.9 |
| Maximum | 118.8 |
| Missing | 34/75 (45) |
| **Pre-bronchodilator FEV1** |  |
| Minimum | 0.5 |
| median (IQR) | 2.0 (1.5, 2.4) |
| mean (sd) | 2.0 ± 0.7 |
| Maximum | 4.0 |
| Missing | 0/75 (0) |
| **Post-bronchodilator FEV1** |  |
| Minimum | 0.6 |
| median (IQR) | 2.0 (1.5, 2.5) |
| mean (sd) | 2.0 ± 0.7 |
| Maximum | 3.6 |
| Missing | 24/75 (32) |
| **Pre-BD % of the predicted FEV1 value** |  |
| Minimum | 27.0 |
| median (IQR) | 78.4 (67.6, 91.4) |
| mean (sd) | 77.9 ± 20.7 |
| Maximum | 118.0 |
| Missing | 1/75 (1) |
| **Post-BD % of the predicted FEV1 value** |  |
| Minimum | 32.0 |
| median (IQR) | 80.0 (72.0, 94.0) |
| mean (sd) | 80.8 ± 18.9 |
| Maximum | 118.0 |
| Missing | 34/75 (45) |
| **Pre-bronchodilator FEV1 / FVC** |  |
| Minimum | 39.7 |
| median (IQR) | 77.0 (70.4, 83.3) |
| mean (sd) | 76.3 ± 11.2 |
| Maximum | 111.3 |
| Missing | 0/75 (0) |
| **Post-bronchodilator FEV1 / FVC** |  |
| Minimum | 41.1 |
| median (IQR) | 78.5 (74.1, 84.1) |
| mean (sd) | 76.7 ± 10.9 |
| Maximum | 91.7 |
| Missing | 24/75 (32) |

SD=Standard deviation; IQR=Interquartile range (25th Percentile to 75th Percentile);
for category variables, n (%) was used for statistics.

## Table 3.4 Blood Test at Visit (in visit window)

| Blood Test | SAU (N = 101) |
| --- | --- |
| **White blood cell count, cells/mm^3^** |  |
| minimum | 3,820.0 |
| median (IQR) | 7,300.0 (6,100.0, 8,910.0) |
| mean (sd) | 7,837.8 ± 2,573.7 |
| maximum | 17,730.0 |
| **Eosinophils%** |  |
| minimum | 0.0 |
| median (IQR) | 3.3 (1.7, 6.3) |
| mean (sd) | 4.7 ± 4.0 |
| maximum | 15.0 |
| **Absolute Eosinophils, cells/mm^3^** |  |
| minimum | 0.0 |
| median (IQR) | 250.0 (110.0, 500.0) |
| mean (sd) | 361.0 ± 344.2 |
| maximum | 1,500.0 |
| **Total serum IgE, IU/ml** |  |
| minimum | 0.0 |
| median (IQR) | 99.5 (38.0, 289.0) |
| mean (sd) | 255.4 ± 430.7 |
| maximum | 2,876.0 |

SD=Standard deviation; IQR=Interquartile range (25th Percentile to 75th Percentile);
for category variables, n (%) was used for statistics.

## Table 3.5 Phenotype

| Phenotype | SAU (N = 101) |
| --- | --- |
| **Eosinophilic phenotype** |  |
| blood eosinophil count ≥300 cells/mm^3^ | 45 (45) |
| **Atopic phenotype** |  |
| total serum IgE > 100 IU/ml | 50 (50) |
| **Overlap of atopic and eosinophilic phenotype** |  |
| total serum IgE > 100 IU/ml & blood eosinophil count ≥ 300 cells/mm^3^ | 25 (25) |
| **Atopic phenotype** |  |
| total serum IgE > 150 IU/ml | 41 (41) |
| **Overlap of atopic and eosinophilic phenotype** |  |
| total serum IgE > 150 IU/ml & blood eosinophil count ≥ 300 cells/mm3 | 23 (23) |

For category variables, n (%) was used for statistics.

**Eosinophil group**

| Eosinophil group | SAU (N = 101) |
| --- | --- |
| **Eosinophilic group** |  |
| >=300 cells/mm^3^ | 45 (45) |
| 150-299 cells/ mm^3^ | 21 (21) |
| <150 cells/ mm^3^ | 35 (35) |

# 4 ASTHMA SYMPTOM CONTROL

## Table 4.1 Asthma Symptom Control

| Asthma Symptom Control | SAU (N = 101) |
| --- | --- |
| **Level of Asthma Symptom Control** |  |
| Well-controlled | 9 (9) |
| Partly controlled | 22 (22) |
| Uncontrolled | 70 (69) |

For category variables, n (%) was used for statistics.

# 5 EOSINOPHILIC ASTHMA PATIENTS SUB-GROUP ANALYSIS

## Table 5.1 Demographic and Lifestyle Characteristics by Phenotype in SAU

| Demographic and Lifestyle Characteristics | SAU (N = 101) | Eosinophilic phenotype (N = 45) | Atopic phenotype1 (N = 50) | Overlap of atopic and eosinophilic phenotype1 (N = 25) | Atopic phenotype2 (N = 41) | Overlap of atopic and eosinophilic phenotype2 (N = 23) |
| --- | --- | --- | --- | --- | --- | --- |
| **Age** |  |  |  |  |  |  |
| minimum | 17.1 | 23.1 | 17.1 | 23.1 | 17.1 | 23.1 |
| median (IQR) | 49.0 (38.0, 57.4) | 49.0 (44.1, 58.9) | 48.0 (37.8, 57.1) | 49.0 (45.2, 56.4) | 46.3 (37.8, 56.9) | 49.0 (45.5, 56.6) |
| mean (sd) | 48.7 ± 13.2 | 50.3 ± 13.6 | 47.7 ± 12.8 | 48.7 ± 10.5 | 47.3 ± 12.9 | 49.0 ± 10.8 |
| maximum | 77.8 | 77.8 | 74.9 | 66.9 | 74.9 | 66.9 |
| **Age group** |  |  |  |  |  |  |
| <=18 | 1 (1) | 0 (0) | 1 (2) | 0 (0) | 1 (2) | 0 (0) |
| 18-54 | 68 (67) | 28 (62) | 33 (66) | 17 (68) | 27 (66) | 15 (65) |
| >=55 | 32 (32) | 17 (38) | 16 (32) | 8 (32) | 13 (32) | 8 (35) |
| **Gender** |  |  |  |  |  |  |
| Female | 84 (83) | 35 (78) | 39 (78) | 18 (72) | 30 (73) | 16 (70) |
| Male | 17 (17) | 10 (22) | 11 (22) | 7 (28) | 11 (27) | 7 (30) |
| **Height** |  |  |  |  |  |  |
| minimum | 120.0 | 120.0 | 120.0 | 120.0 | 120.0 | 120.0 |
| median (IQR) | 156.0 (151.0, 163.0) | 156.0 (151.0, 165.0) | 156.5 (151.2, 165.0) | 157.0 (156.0, 168.0) | 157.0 (154.0, 165.0) | 156.0 (155.5, 166.5) |
| mean (sd) | 157.1 ± 8.8 | 157.0 ± 10.1 | 157.9 ± 10.0 | 158.7 ± 10.7 | 158.4 ± 10.2 | 158.2 ± 10.8 |
| maximum | 175.0 | 175.0 | 175.0 | 172.0 | 175.0 | 172.0 |
| **Weight** |  |  |  |  |  |  |
| minimum | 30.0 | 30.0 | 30.0 | 30.0 | 30.0 | 30.0 |
| median (IQR) | 80.0 (67.1, 88.9) | 75.0 (64.0, 94.6) | 78.5 (68.2, 92.0) | 84.0 (69.0, 98.0) | 72.5 (67.1, 87.0) | 76.0 (68.0, 95.6) |
| mean (sd) | 79.1 ± 19.2 | 77.6 ± 21.4 | 79.8 ± 19.4 | 82.0 ± 23.6 | 78.1 ± 19.4 | 79.5 ± 22.5 |
| maximum | 150.0 | 125.0 | 125.0 | 125.0 | 125.0 | 125.0 |
| **BMI** |  |  |  |  |  |  |
| minimum | 15.9 | 15.9 | 20.0 | 20.0 | 20.0 | 20.0 |
| median (IQR) | 31.7 (26.2, 36.8) | 29.4 (25.6, 36.8) | 31.8 (26.1, 37.0) | 29.6 (25.7, 39.1) | 28.7 (25.2, 34.9) | 29.3 (25.4, 35.8) |
| mean (sd) | 32.0 ± 7.2 | 31.3 ± 7.7 | 31.9 ± 6.9 | 32.2 ± 7.9 | 31.0 ± 7.0 | 31.4 ± 7.8 |
| maximum | 58.6 | 46.1 | 46.1 | 46.1 | 46.1 | 46.1 |
| **BMI group** |  |  |  |  |  |  |
| <18.5 | 1 (1) | 1 (2) | 0 (0) | 0 (0) | 0 (0) | 0 (0) |
| 18.5-24.9 | 17 (17) | 9 (20) | 9 (18) | 5 (20) | 9 (22) | 5 (22) |
| ≥25-29.9 | 25 (25) | 15 (33) | 15 (30) | 8 (32) | 14 (34) | 8 (35) |
| ≥30 | 58 (57) | 20 (44) | 26 (52) | 12 (48) | 18 (44) | 10 (43) |
| **Educational level** |  |  |  |  |  |  |
| Elementary school | 12 (12) | 4 (9) | 7 (14) | 3 (12) | 4 (10) | 3 (13) |
| Secondary school | 12 (12) | 7 (16) | 6 (12) | 4 (16) | 6 (15) | 4 (17) |
| High-school | 10 (10) | 5 (11) | 6 (12) | 3 (12) | 4 (10) | 1 (4) |
| University and post-university education | 27 (27) | 15 (33) | 15 (30) | 10 (40) | 14 (34) | 10 (43) |
| Unknown | 40 (40) | 14 (31) | 16 (32) | 5 (20) | 13 (32) | 5 (22) |
| **Insurance status** |  |  |  |  |  |  |
| Fully Reimbursed | 69 (68) | 27 (60) | 31 (62) | 12 (48) | 24 (59) | 11 (48) |
| Partially Reimbursed | 7 (7) | 3 (7) | 5 (10) | 3 (12) | 5 (12) | 3 (13) |
| Not reimbursed | 24 (24) | 15 (33) | 13 (26) | 10 (40) | 11 (27) | 9 (39) |
| Unknown | 1 (1) | 0 (0) | 1 (2) | 0 (0) | 1 (2) | 0 (0) |
| **Smoking status history** |  |  |  |  |  |  |
| Former smoker | 9 (9) | 6 (13) | 5 (10) | 4 (16) | 4 (10) | 3 (13) |
| Never smoker | 92 (91) | 39 (87) | 45 (90) | 21 (84) | 37 (90) | 20 (87) |

*Atopic phenotype1: total serum IgE > 100 IU/ml,*

*Overlap of atopic and eosinophilic phenotype1: total serum IgE > 100 IU/ml & blood eosinophil count ≥ 300 cells/mm3,*

*Atopic phenotype2: total serum IgE > 150 IU/ml,*

*Overlap of atopic and eosinophilic phenotype2: total serum IgE > 150 IU/ml & blood eosinophil count ≥ 300 cells/mm3,*

*SD=Standard deviation; IQR=Interquartile range (25th Percentile to 75th Percentile);
for category variables, n (%) was used for statistics.*

## Table 5.2 Disease Characteristics by Phenotype in SAU

| Disease Characteristics | SAU (N = 101) | Eosinophilic phenotype (N = 45) | Atopic phenotype1 (N = 50) | Overlap of atopic and eosinophilic phenotype1 (N = 25) | Atopic phenotype2 (N = 41) | Overlap of atopic and eosinophilic phenotype2 (N = 23) |
| --- | --- | --- | --- | --- | --- | --- |
| **Asthma_duration** |  |  |  |  |  |  |
| minimum | 1.0 | 1.5 | 1.0 | 1.5 | 1.0 | 2.6 |
| median (IQR) | 15.9 (8.3, 24.7) | 17.8 (7.8, 26.9) | 13.4 (6.6, 22.9) | 15.0 (6.5, 23.0) | 14.0 (7.1, 24.9) | 15.0 (6.8, 24.4) |
| mean (sd) | 17.6 ± 12.1 | 19.4 ± 12.8 | 15.6 ± 11.5 | 16.5 ± 12.7 | 16.6 ± 12.1 | 17.2 ± 12.9 |
| maximum | 57.8 | 54.9 | 54.9 | 54.9 | 54.9 | 54.9 |
| **Number of severe asthma exacerbations in the last year** |  |  |  |  |  |  |
| minimum | 0.0 | 0.0 | 0.0 | 0.0 | 0.0 | 0.0 |
| median (IQR) | 2.0 (1.0, 3.0) | 2.0 (1.0, 3.0) | 2.0 (1.0, 4.0) | 2.0 (1.0, 3.0) | 2.0 (1.0, 4.0) | 2.0 (1.0, 2.5) |
| mean (sd) | 2.7 ± 4.2 | 2.4 ± 3.4 | 3.2 ± 4.0 | 2.9 ± 3.9 | 3.3 ± 4.3 | 3.0 ± 4.0 |
| maximum | 30.0 | 20.0 | 20.0 | 20.0 | 20.0 | 20.0 |
| **Number of severe asthma exacerbations in the last year by groups** |  |  |  |  |  |  |
| 0 | 24 (24) | 9 (20) | 7 (14) | 3 (12) | 5 (12) | 2 (9) |
| 1 | 21 (21) | 11 (24) | 10 (20) | 5 (20) | 8 (20) | 5 (22) |
| 2 | 24 (24) | 12 (27) | 15 (30) | 10 (40) | 13 (32) | 10 (43) |
| 3 | 9 (9) | 6 (13) | 2 (4) | 1 (4) | 2 (5) | 1 (4) |
| >3 | 23 (23) | 7 (16) | 16 (32) | 6 (24) | 13 (32) | 5 (22) |
| **Asthma classification** |  |  |  |  |  |  |
| Early-onset asthma: Asthma diagnosed at <12 years of age. | 14 (14) | 9 (20) | 5 (10) | 4 (16) | 5 (12) | 4 (17) |
| Late-onset asthma: Asthma diagnosed ≥12 years of age. | 87 (86) | 36 (80) | 45 (90) | 21 (84) | 36 (88) | 19 (83) |
| **History of atopy** |  |  |  |  |  |  |
| NO | 83 (82) | 35 (78) | 38 (76) | 16 (64) | 30 (73) | 15 (65) |
| YES | 18 (18) | 10 (22) | 12 (24) | 9 (36) | 11 (27) | 8 (35) |
| **Skin prick test history** |  |  |  |  |  |  |
| NO | 39 (98) | 16 (100) | 18 (100) | 11 (100) | 14 (100) | 10 (100) |
| YES | 1 (2) | 0 (0) | 0 (0) | 0 (0) | 0 (0) | 0 (0) |
| Missing | 61/101 (60) | 29/45 (64) | 32/50 (64) | 14/25 (56) | 27/41 (66) | 13/23 (57) |
| **If yes skin prick test** |  |  |  |  |  |  |
| Positive | 1 (100) | 0 (NaN) | 0 (NaN) | 0 (NaN) | 0 (NaN) | 0 (NaN) |
| **Aeroallergens positive** |  |  |  |  |  |  |
| N/A | 1 (100) | 0 (NaN) | 0 (NaN) | 0 (NaN) | 0 (NaN) | 0 (NaN) |
| Missing | 100/101 (99) | 45/45 (100) | 50/50 (100) | 25/25 (100) | 41/41 (100) | 23/23 (100) |
| **Specific IgE available in the medical records** |  |  |  |  |  |  |
| NO | 3 (7) | 2 (12) | 1 (5) | 1 (9) | 1 (7) | 1 (10) |
| YES | 38 (93) | 14 (88) | 18 (95) | 10 (91) | 14 (93) | 9 (90) |
| Missing | 60/101 (59) | 29/45 (64) | 31/50 (62) | 14/25 (56) | 26/41 (63) | 13/23 (57) |
| **If yes specific IgE** |  |  |  |  |  |  |
| Negative | 20 (53) | 5 (36) | 4 (22) | 2 (20) | 2 (14) | 1 (11) |
| Positive | 18 (47) | 9 (64) | 14 (78) | 8 (80) | 12 (86) | 8 (89) |
| **Comorbidities** |  |  |  |  |  |  |
| NO | 35 (35) | 21 (48) | 20 (40) | 14 (56) | 18 (44) | 13 (57) |
| YES | 65 (65) | 23 (52) | 30 (60) | 11 (44) | 23 (56) | 10 (43) |
| Missing | 1/101 (1) | 1/45 (2) | 0/50 (0) | 0/25 (0) | 0/41 (0) | 0/23 (0) |
| **Co-morbidity type** | 65 | 23 | 30 | 11 | 23 | 10 |
| Rhinitis | 42 (64.6) | 16 (69.6) | 23 (76.7) | 9 (81.8) | 19 (82.6) | 9 (90.0) |
| Nasal polyps | 17 (26.2) | 7 (30.4) | 11 (36.7) | 5 (45.5) | 9 (39.1) | 5 (50.0) |
| Atopic dermatitis | 2 (3.1) | 0 (0.0) | 1 (3.3) | 0 (0.0) | 1 (4.3) | 0 (0.0) |
| Asthma associated with NSAIDs | 0 (0.0) | 0 (0.0) | 0 (0.0) | 0 (0.0) | 0 (0.0) | 0 (0.0) |
| Sinusitis | 14 (21.5) | 4 (17.4) | 8 (26.7) | 3 (27.3) | 6 (26.1) | 3 (30.0) |
| Aspergillosis | 0 (0.0) | 0 (0.0) | 0 (0.0) | 0 (0.0) | 0 (0.0) | 0 (0.0) |
| Eczema | 1 (1.5) | 0 (0.0) | 0 (0.0) | 0 (0.0) | 0 (0.0) | 0 (0.0) |
| Urticaria | 0 (0.0) | 0 (0.0) | 0 (0.0) | 0 (0.0) | 0 (0.0) | 0 (0.0) |

*Atopic phenotype1: total serum IgE > 100 IU/ml,*

*Overlap of atopic and eosinophilic phenotype1: total serum IgE > 100 IU/ml & blood eosinophil count ≥ 300 cells/mm3,*

*Atopic phenotype2: total serum IgE > 150 IU/ml,*

*Overlap of atopic and eosinophilic phenotype2: total serum IgE > 150 IU/ml & blood eosinophil count ≥ 300 cells/mm3,*

*SD=Standard deviation; IQR=Interquartile range (25th Percentile to 75th Percentile);
for category variables, n (%) was used for statistics.*

## Table 5.3 Pattern of CS Use by Phenotype in SAU

| Phenotype | SAU (N = 101) | CS burst treatment/  Short Course (N = 60) | Chronic OCS (N = 7) |
| --- | --- | --- | --- |
| **Eosinophilic phenotype** |  |  |  |
| blood eosinophil count ≥300 cells/mm3 | 45 (45) | 29 (48) | 3 (43) |
| **Atopic phenotype1** |  |  |  |
| total serum IgE > 100 IU/ml | 50 (50) | 32 (53) | 2 (29) |
| **Overlap of atopic and eosinophilic phenotype1** |  |  |  |
| total serum IgE > 100 IU/ml & blood eosinophil count ≥ 300 cells/mm3 | 25 (25) | 16 (27) | 1 (14) |
| **Atopic phenotype2** |  |  |  |
| total serum IgE > 150 IU/ml | 41 (41) | 28 (47) | 2 (29) |
| **Overlap of atopic and eosinophilic phenotype2** |  |  |  |
| total serum IgE > 150 IU/ml & blood eosinophil count ≥ 300 cells/mm3 | 23 (23) | 16 (27) | 1 (14) |

*#For category variables, n (%) was used for statistics.*

| CS Use | SAU (N = 101) | Eosinophilic phenotype (N = 45) | Atopic phenotype1 (N = 50) | Overlap of atopic and eosinophilic phenotype1 (N = 25) | Atopic phenotype2 (N = 41) | Overlap of atopic and eosinophilic phenotype2 (N = 23) |
| --- | --- | --- | --- | --- | --- | --- |
| **CS burst treatment/short course** |  |  |  |  |  |  |
| Yes | 60 (60) | 29 (64) | 32 (65) | 16 (64) | 28 (70) | 16 (70) |
| **Chronic OCS** |  |  |  |  |  |  |
| Yes | 7 (7) | 3 (7) | 2 (4) | 1 (4) | 2 (5) | 1 (4) |

*Atopic phenotype1: total serum IgE > 100 IU/ml,*

*Overlap of atopic and eosinophilic phenotype1: total serum IgE > 100 IU/ml & blood eosinophil count ≥ 300 cells/mm3,*

*Atopic phenotype2: total serum IgE > 150 IU/ml,*

*Overlap of atopic and eosinophilic phenotype2: total serum IgE > 150 IU/ml & blood eosinophil count ≥ 300 cells/mm3,*

## Table 5.4 Chi-square Test of Association between Asthma Control Levels and Phenotype in SAU

| Phenotype | SAU (N = 101) | Well-controlled (N = 9) | Partly controlled (N = 22) | Uncontrolled (N = 70) | P value* |
| --- | --- | --- | --- | --- | --- |
| **Eosinophilic phenotype** |  |  |  |  |  |
| blood eosinophil count ≥300 cells/mm3 | 45 (45) | 2 (22) | 11 (50) | 32 (46) | 0.347 |
| **Atopic phenotype1** |  |  |  |  |  |
| total serum IgE > 100 IU/ml | 50 (50) | 5 (56) | 13 (59) | 32 (46) | 0.511 |
| **Overlap of atopic and eosinophilic phenotype1** |  |  |  |  |  |
| total serum IgE > 100 IU/ml & blood eosinophil count ≥ 300 cells/mm3 | 25 (25) | 2 (22) | 7 (32) | 16 (23) | 0.685 |
| **Atopic phenotype2** |  |  |  |  |  |
| total serum IgE > 150 IU/ml | 41 (41) | 3 (33) | 10 (45) | 28 (40) | 0.810 |
| **Overlap of atopic and eosinophilic phenotype2** |  |  |  |  |  |
| total serum IgE > 150 IU/ml & blood eosinophil count ≥ 300 cells/mm3 | 23 (23) | 2 (22) | 6 (27) | 15 (21) | 0.849 |

*#For category variables, n (%) was used for statistics.*

*chi-square test of Each phenotype distribution (Yes/No) cross asthma control levels.

| Asthma Control Levels | SAU (N = 101) | Eosinophilic phenotype (N = 45) | Atopic phenotype1 (N = 50) | Overlap of atopic and eosinophilic phenotype1 (N = 25) | Atopic phenotype2 (N = 41) | Overlap of atopic and eosinophilic phenotype2 (N = 23) |
| --- | --- | --- | --- | --- | --- | --- |
| **Level of Asthma Symptom Control** |  |  |  |  |  |  |
| Well-controlled | 9 (9) | 2 (4) | 5 (10) | 2 (8) | 3 (7) | 2 (9) |
| Partly controlled | 22 (22) | 11 (24) | 13 (26) | 7 (28) | 10 (24) | 6 (26) |
| Uncontrolled | 70 (69) | 32 (71) | 32 (64) | 16 (64) | 28 (68) | 15 (65) |

*Atopic phenotype1: total serum IgE > 100 IU/ml,*

*Overlap of atopic and eosinophilic phenotype1: total serum IgE > 100 IU/ml & blood eosinophil count ≥ 300 cells/mm3,*

*Atopic phenotype2: total serum IgE > 150 IU/ml,*

*Overlap of atopic and eosinophilic phenotype2: total serum IgE > 150 IU/ml & blood eosinophil count ≥ 300 cells/mm3,*

### Chi-square Test of Association between Asthma Control Levels and Phenotype in SAU (atopic phenotype as total serum IgE > 100 IU/ml)

|  | SAU | Well-controlled | Partly controlled | Uncontrolled | p |
| --- | --- | --- | --- | --- | --- |
| n | 70 | 5 | 17 | 48 |  |
| Phenotype (%) |  |  |  |  | 0.540 |
| Only atopic phenotype1 | 25 (35.7) | 3 (60.0) | 6 (35.3) | 16 (33.3) |  |
| Only eosinophilic phenotype1 | 20 (28.6) | 0 (0.0) | 4 (23.5) | 16 (33.3) |  |
| Overlap of atopic and eosinophilic phenotype1 | 25 (35.7) | 2 (40.0) | 7 (41.2) | 16 (33.3) |  |

Chi-square Test of Association between Asthma Control Levels and Phenotype in SAU (atopic phenotype as total serum IgE > 100 IU/ml)

|  | SAU | Only atopic phenotype1 | Only eosinophilic phenotype1 | Overlap of atopic and eosinophilic phenotype1 | p |
| --- | --- | --- | --- | --- | --- |
| n | 70 | 25 | 20 | 25 |  |
| Level of Asthma Symptom Control (%) |  |  |  |  | 0.540 |
| Well-controlled | 5 (7.1) | 3 (12.0) | 0 (0.0) | 2 (8.0) |  |
| Partly controlled | 17 (24.3) | 6 (24.0) | 4 (20.0) | 7 (28.0) |  |
| Uncontrolled | 48 (68.6) | 16 (64.0) | 16 (80.0) | 16 (64.0) |  |

*For category variables, n (%) was used for statistics.*

*n：blood eosinophil count ≥300 cells/mm3 or total serum IgE > 100 IU/ml.*

*Only atopic phenotype1: total serum IgE >100 IU/ml and blood eosinophil count <300 cells/mm^3^.*

*Only eosinophilic phenotype1: blood eosinophil count ≥300 cells/mm^3^ and total serum IgE <= 100 IU/ml.*

*Overlap of atopic and eosinophilic phenotype1: blood eosinophil count ≥300 cells/mm^3^ and total serum IgE > 100 IU/ml.*

### Chi-square Test of Association between Asthma Control Levels and Phenotype in SAU (atopic phenotype as total serum IgE > 150 IU/ml)

|  | SAU | Well-controlled | Partly controlled | Uncontrolled | p |
| --- | --- | --- | --- | --- | --- |
| n | 63 | 3 | 15 | 45 |  |
| Phenotype (%) |  |  |  |  | 0.712 |
| Only atopic phenotype2 | 18 (28.6) | 1 (33.3) | 4 (26.7) | 13 (28.9) |  |
| Only eosinophilic phenotype2 | 22 (34.9) | 0 (0.0) | 5 (33.3) | 17 (37.8) |  |
| Overlap of atopic and eosinophilic phenotype2 | 23 (36.5) | 2 (66.7) | 6 (40.0) | 15 (33.3) |  |

Chi-square Test of Association between Asthma Control Levels and Phenotype in SAU (atopic phenotype as total serum IgE > 150 IU/ml)

|  | SAU | Only atopic phenotype2 | Only eosinophilic phenotype2 | Overlap of atopic and eosinophilic phenotype2 | p |
| --- | --- | --- | --- | --- | --- |
| n | 63 | 18 | 22 | 23 |  |
| Level of Asthma Symptom Control (%) |  |  |  |  | 0.712 |
| Well-controlled | 3 (4.8) | 1 (5.6) | 0 (0.0) | 2 (8.7) |  |
| Partly controlled | 15 (23.8) | 4 (22.2) | 5 (22.7) | 6 (26.1) |  |
| Uncontrolled | 45 (71.4) | 13 (72.2) | 17 (77.3) | 15 (65.2) |  |

*For category variables, n (%) was used for statistics.*

*n：blood eosinophil count ≥300 cells/mm3 or total serum IgE > 150 IU/ml.*

*Only atopic phenotype2: total serum IgE >150 IU/ml and blood eosinophil count <300 cells/mm^3^.*

*Only eosinophilic phenotype2: blood eosinophil count ≥300 cells/mm^3^ and total serum IgE <= 150 IU/ml.*

*Overlap of atopic and eosinophilic phenotype2: blood eosinophil count ≥300 cells/mm^3^ and total serum IgE > 150 IU/ml.*

Chi-square Test of Association between Asthma Control Levels and Phenotype in SAU (atopic phenotype as total serum IgE > 150 IU/ml)

|  | SAU | Well-controlled | Partly controlled | Uncontrolled | p |
| --- | --- | --- | --- | --- | --- |
| n | 63 | 3 | 15 | 45 |  |
| Phenotype (%) |  |  |  |  | 0.712 |
| Only atopic phenotype | 18 (28.6) | 1 (33.3) | 4 (26.7) | 13 (28.9) |  |
| Only eosinophilic phenotype | 22 (34.9) | 0 (0.0) | 5 (33.3) | 17 (37.8) |  |
| Overlap of atopic and eosinophilic phenotype | 23 (36.5) | 2 (66.7) | 6 (40.0) | 15 (33.3) |  |

## Figure 5.6 ANOVA Test of Differences in Number of Severe Exacerbations across Phenotype in SAU (atopic phenotype as total serum IgE > 100 IU/ml)


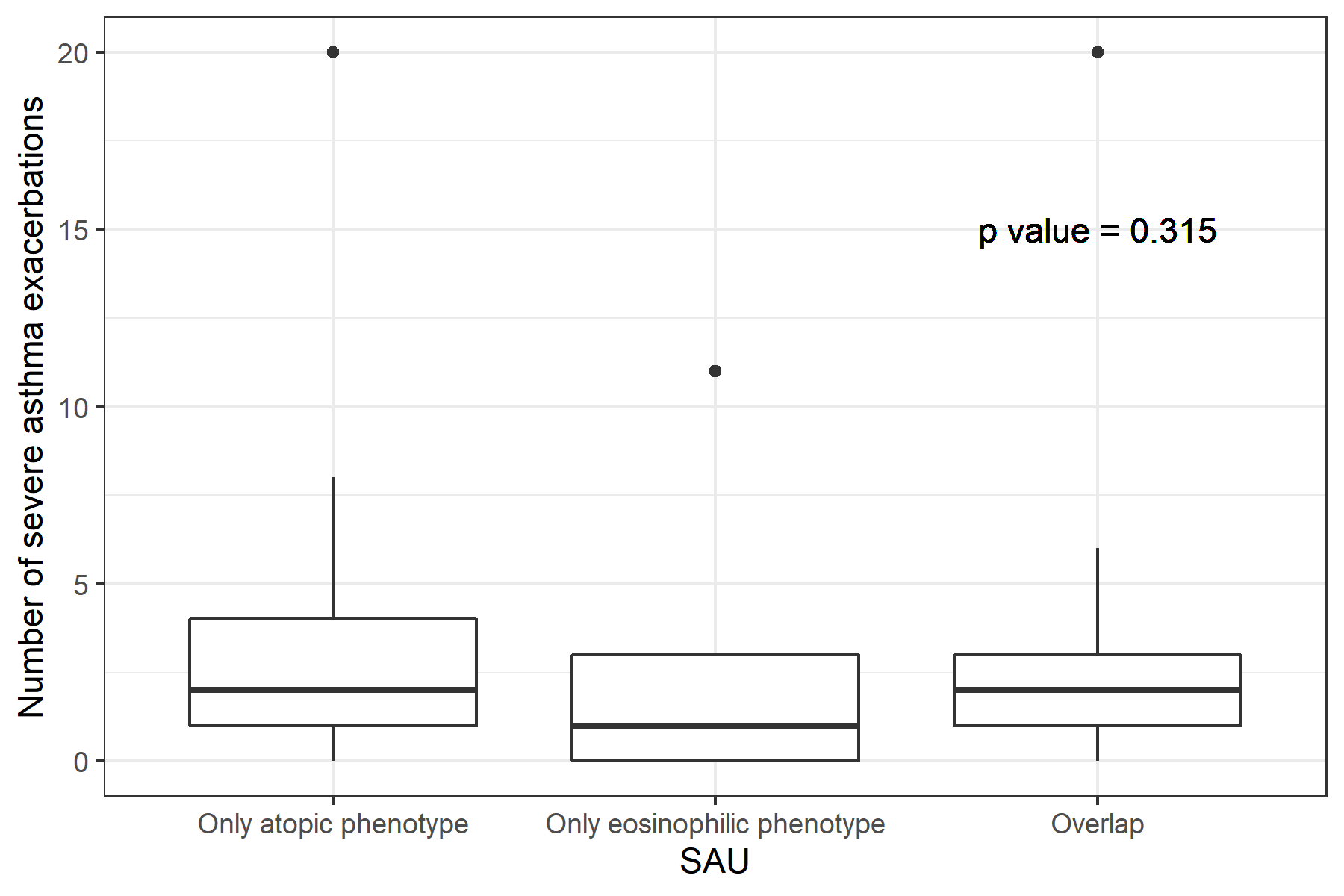


* Only atopic phenotype: total serum IgE >100 IU/ml and blood eosinophil count <300 cells/mm^3^.

Only eosinophilic phenotype: blood eosinophil count ≥300 cells/mm^3^ and total serum IgE <= 100 IU/ml.

Overlap: overlap of atopic and eosinophilic phenotype as blood eosinophil count ≥300 cells/mm^3^ and total serum IgE > 100 IU/ml.

## ANOVA Test of Differences in Number of Severe Exacerbations across Phenotype in SAU (atopic phenotype as total serum IgE > 150 IU/ml)


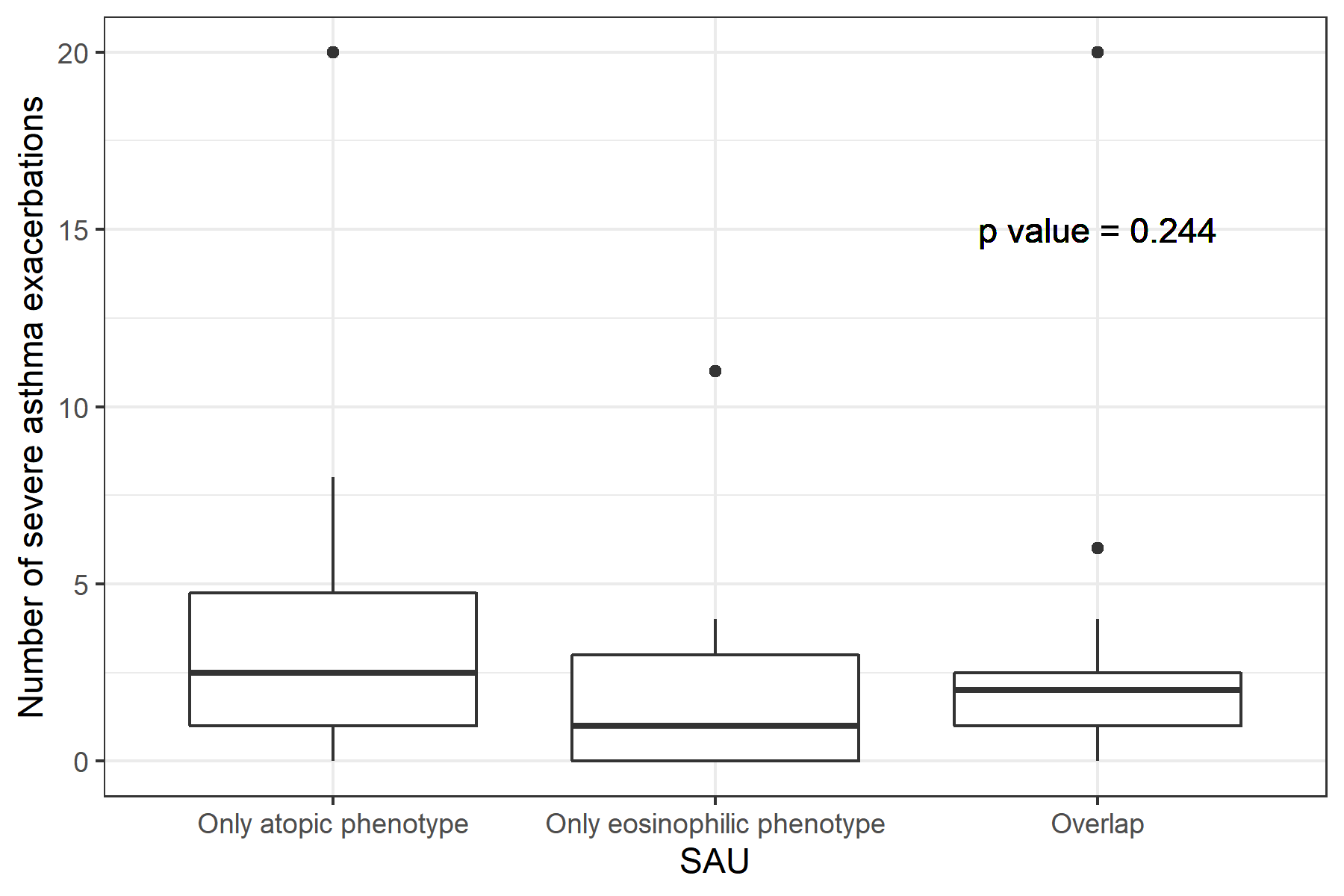


*Only atopic phenotype: total serum IgE >150 IU/ml and blood eosinophil count <300 cells/mm3.

Only eosinophilic phenotype: blood eosinophil count ≥300 cells/mm^3^ and total serum IgE <= 150 IU/ml.

Overlap: overlap of atopic and eosinophilic phenotype as blood eosinophil count ≥300 cells/mm^3^ and total serum IgE > 150 IU/ml.
